# Supplementary material for: The Evolution and Characterization of the RNA Interference Pathways in Lophotrochozoa
Source: Genome Biol Evol. 2024 May 7;16(5):evae098. doi: 10.1093/gbe/evae098 (PMC11114477; doi:10.1093/gbe/evae098)
Supplement: evae098_Supplementary_Data [file evae098_supplementary_data.zip › Supplementary_materials.zip!/selected_miRNAs/NoGe_selected_miRNAs/known_miRNA_families_pdf_files/NMRB01000494.1_Notospermus_geniculatus_strain_Ushimado_scaffold494,_whole_genome_shotgun_sequence_14536.pdf]

Provisional ID	:	NMRB01000494.1_Notospermus_geniculatus_strain_Ushimado_scaffold494,_whole_genome_shotgun_sequence_14536
Score total	:	111586.6
Score for star read(s)	:	3.9
Score for read counts	:	111577.9
Score for mfe	:	1.8
Score for randfold	:	0
Score for cons. seed	:	3
Total read count	:	218867
Mature read count	:	218592
Loop read count	:	0
Star read count	:	275

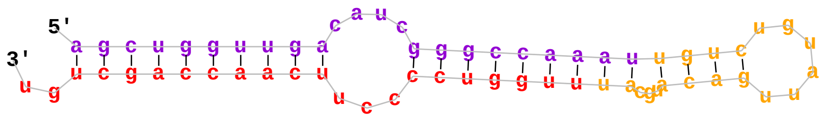

Genome coordinates: NMRB01000494.1 83039-83103

pre-miRNA sequence:
AGCUGGUUGACAUCGGGCCAAAUAGCUGGUUGACAUCGGGCCAAAUUGUCUGUAUUGACAUGCAUUUGGUCCCCUUCAACCAGCUGU

Mature sequence: UUGGUCCCCUUCAACCAGCUGU

Aligned mature sequence of the *N. geniculatus* pre-miRNA with the top 5 hits from the BLAST search against the pre-miRNA mirGeneDB set:

```
                                   **********************
     Nge_Mir-133                   uugguccccuucaaccagcugu
     Npo-Mir-133-P20_3p            uugguccccuucaaccagcugu
     Npo-Mir-133-P21_3p            uugguccccuucaaccagcugu
     Obi-Mir-133_3p                uugguccccuucaaccagcugu
     Ovu-Mir-133_3p                uugguccccuucaaccagcugu
     Lan-Mir-133_3p                uugguccccuucaaccagcugu
```
